# Successful redo aortic valve replacement using Perceval for multiple recurrent prosthetic valve dysfunction

**DOI:** 10.1093/icvts/ivab361

**Published:** 2022-02-16

**Authors:** Yumi Kakizawa, Hiroyuki Nishi, Takasumi Goto, Mutsunori Kitahara

**Affiliations:** Department of Cardiovascular Surgery, Osaka General Medical Center, Osaka, Japan

**Keywords:** Paravalvular leakage, Redo aortic valve replacement, Sutureless valve

## Abstract

A 50-year-old man who required aortic valve replacement (AVR) 3 times for severe paravalvular leakage (PVL) was diagnosed with a recurrence of severe PVL. Since the PVL recurred each time because of prosthetic valve detachment from the annulus, a fourth AVR was performed using a self-expanding sutureless valve. Postoperative echocardiography showed no significant PVL. The patient was discharged without any complications and returned to normal work soon after. In cases such as our patient, sutureless AVR could be a powerful alternative to conventional AVR.

## INTRODUCTION

Sutureless aortic valve replacement (SU-AVR) is an attractive surgical option facilitated by the recent advances in aortic valve device technology [1]. Since SU-AVR alleviates the need for suturing and allows for quick implantation, it can reduce operation time significantly. Furthermore, since Perceval implantation does not require any annular sutures, SU-AVR using a Perceval might provide additional advantages in patients with complicated recurrence of paravalvular leakage (PVL) due to annular suture failure.

Here, we report a fourth successful redo-AVR using a Perceval for frequent recurrences of PVL after conventional AVR.

## PRESENTATION OF CASE

A 50-year-old man who had dyspnoea on effort was referred to our institution for surgical treatment of recurring severe PVL after AVR. The patient had undergone AVR 3 times previously. Initial AVR (Björk–Shiley, 25 mm, Pfizer Inc., USA) was performed for severe aortic regurgitation 38 years prior. A second AVR (Björk–Shiley, 25 mm, Pfizer Inc., USA) was performed 34 years prior because of annular suture failure. The third AVR (ATS, 25 mm, ATS Medical, Inc., USA) was performed 18 years ago (Supplementary Material, Fig. S1).

**Figure 1:**
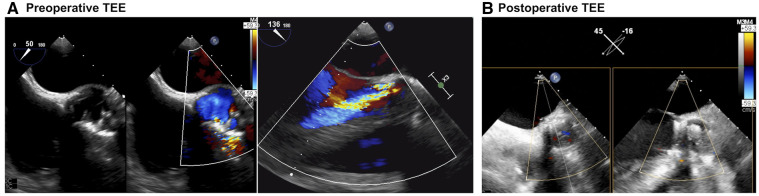
Preoperative and postoperative echocardiogram. (**A**) Preoperative transesophageal echocardiography (TEE) showed severe paravalvular leakage (PVL) from some aortic annular sutures at the right sinus of Valsalva. (**B**) No significant PVL was observed postoperatively. Each echocardiogram is shown as a Video 1.

On echocardiography, the left ventricular ejection fraction was 40%, and the left ventricular end-diastolic and end-systolic diameters were 62 and 50 mm, respectively. Echocardiography demonstrated severe PVL located at the right sinus of Valsalva (Fig. 1A). Thus, the patient was diagnosed with a recurrence of PVL caused by annular suture failure. Since all past redo-AVRs had been performed for PVL caused by annular suture detachment, the potential risk of PVL recurrence could have been higher when performing the fourth redo-AVR using a conventional valve. In addition, the operating time would be longer than the previous redo-AVRs due to dense adherence. Thus, we planned to perform fourth redo-AVR using a Perceval (LivaNova, London, UK).

**Figure 2:**
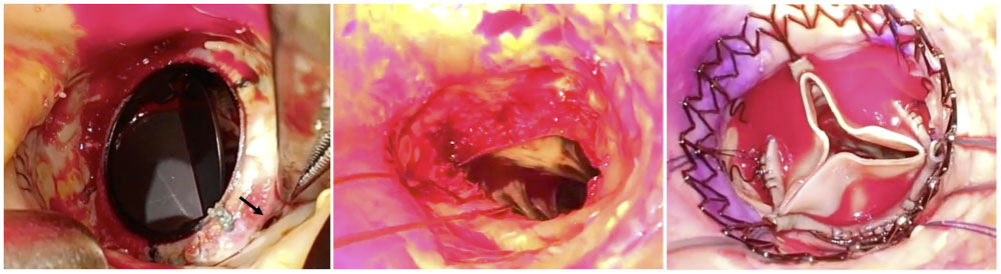
Perioperative findings. Failure of the aortic annular sutures at the right sinus of Valsalva (arrow). After complete removal of the prosthesis, Perceval was implanted successfully. We checked the fitting of the Perceval into the annulus using a video camera.

Since the prosthetic pericardial sheet covered the heart during the third redo-AVR and there was relatively a gap between the sternum and the right heart in preoperative computed tomography, the fourth resternotomy could be performed relatively easy without a tight adhesion. Cardiac arrest was established under the usual cardiopulmonary bypass with normal tepid temperature. A higher transverse aortotomy was performed compared with the past aortotomy, and the prosthetic valve was exposed; there were some gaps between the prosthetic valve and the right sinus of Valsalva. After complete removal of the prosthesis, the sizer of L size Perceval could not pass through the annulus easily. So, Perceval (size M) was implanted precisely by using 3 guiding sutures through the nadir of each cusp (Fig. 2). Intraoperative TEE showed no evidence of PVL (Fig. 1B and Video 1).

The postoperative course was uneventful. Postoperative echocardiography showed that the aortic valve area was 1.82 cm^2^ and the mean pressure gradient was 12 mmHg. The patient was discharged without any complications and returned to normal work soon after.

## DISCUSSION

In the present case, PVL recurred 3 times due to annular suture detachment. This is a common complication in those who have Takayasu arteritis and Behcet’s disease [2]. Our patient did not have such immunological backgrounds, and histological analysis of the aorta showed no evidence of cystic medial necrosis and invasion of inflammatory cells (Supplementary material, Fig.S2). Since initial AVR was performed for bicuspid aortic valve, it might have caused tissue fragility of the aortic annulus. Or, there might have been over-resection of the valve remnant at the initial AVR. Given the patient’s medical history, the potential risk of PVL recurrence was higher in our case than in usual cases. Since annular suture could be a risk factor of PVL recurrence, redo-AVR using Perceval might be more suitable than redo-AVR using conventional valve because Perceval is a self-expanding valve. Moreover, the mid-term outcome of PVL after Perceval implantation is reportedly better than conventional AVR [3]. To the best of our knowledge, our case is the first to report redo-SU-AVR for recurrence of PVL by annular suture detachment.

The surgical indication of AVR with bioprosthesis for relatively younger patients remains controversial. The effective orifice area of Perceval is generally larger than that of conventional bioprosthetic valves [4]. Therefore, valve-in-valve transcatheter aortic valve implantation is more suitable for SU-AVR than traditional AVR. Although the dilatation of the aortic annulus was not significant thus far, it may enlarge in the future, leading to PVL recurrence. PVL occurs outside of the valve; therefore, valve-in-valve transcatheter aortic valve implantation is not suitable for patients undergoing conventional AVR or SU-AVR using Intuity (Edwards Lifesciences, Irvine, CA, USA). However, since Perceval is a self-expanding valve, future valve-in-valve transcatheter aortic valve implantation can be theoretically performed in Perceval implantation cases. Given these reasons, SU-AVR with Perceval can be an acceptable surgical option in such cases.

## CONCLUSION

In summary, redo-AVR using Perceval could be a powerful surgical alternative to conventional redo-AVR, especially in patients having a recurrence of PVL caused by the annular anastomosis detachment.

## SUPPLEMENTARY MATERIAL

Supplementary material is available at *ICVTS* online.

## Supplementary Material

ivab361_Supplementary_Data
