# Supplementary material for: Successful redo aortic valve replacement using Perceval for multiple recurrent prosthetic valve dysfunction
Source: Interact Cardiovasc Thorac Surg. 2022 Feb 16;34(5):921–3. doi: 10.1093/icvts/ivab361 (PMC9153371; doi:10.1093/icvts/ivab361)
Supplement: ivab361_Supplementary_Data [file ivab361_Supplementary_Data.docx]

**Supplementary files**

Supplemental Figure 1: location of the paravalvular leak

The schema demonstratins the location of paravalvular leaks in each redo-AVR procedure.

LCA; left coronary artery, RCA; right coronary artery

Supplemental Figure 2: Histological analysis of aortic wall

A: No inflammatory reactions such as lymphocytes or monocytes invasion (Hematoxylin-Eosin stain, x40/x400) were observed. B: There was no evidence suggesting degeneration of the elastic fiber (Elastica-colloidal iron stain, x40).


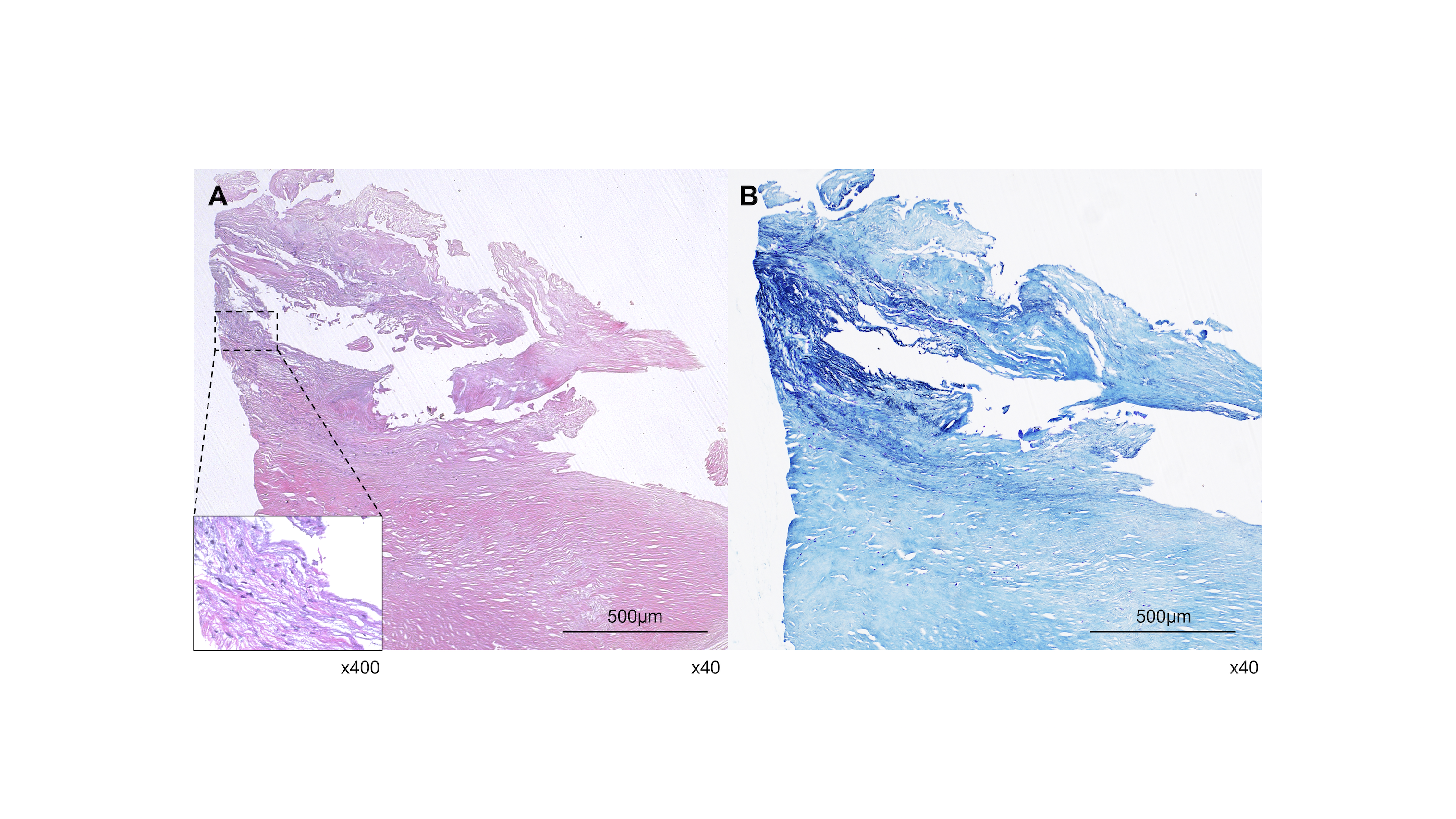


Supplemental Movie: Preoperative and postoperative echocardiogram
